# Trends in uncomplicated and severe malaria following seasonal malaria chemoprevention administration in Nouna, Burkina Faso: a quasi-experimental pre-post study

**DOI:** 10.1186/s12936-025-05597-y

**Published:** 2025-11-06

**Authors:** Elisabeth A. Gebreegziabher, Mamadou Ouattara, Mamadou Bountogo, Boubacar Coulibaly, Valentin Boudo, Thierry Ouedraogo, Elodie Lebas, Huiyu Hu, David V. Glidden, Benjamin F. Arnold, Thomas M. Lietman, Ali Sié, Catherine E. Oldenburg

**Affiliations:** 1 Francis I. Proctor Foundation, University of California, San Francisco, San Francisco, CA USA; 2 Centre de Recherche en Sante de Nouna, Nouna, Burkina Faso; 3 Department of Epidemiology and Biostatistics, University of California, San Francisco, CA USA; 4 Department of Ophthalmology, University of California, San Francisco, San Francisco, CA USA

**Keywords:** Seasonal malaria chemoprevention, Malaria

## Abstract

**Background:**

While Seasonal Malaria Chemoprevention (SMC) has been adopted as a malaria control strategy in regions with seasonal transmission, continued monitoring and evaluation of its effectiveness across diverse ecological, epidemiological, and healthcare settings remain critical for optimizing the intervention. This study aims to assess the ongoing population-level impact of SMC under routine programme conditions by evaluating rates of uncomplicated and severe malaria following four rounds of administration.

**Methods:**

A pre-post analysis was conducted using real-world surveillance data from clinic visits in 285 villages in Nouna District, Burkina Faso, along with National Malaria Control Programme data on SMC administration. Estimates of the population used for person-time calculations were derived from a census conducted as part of a randomized controlled trial. Malaria rates for children under 5 were analyzed for each epidemiological week in 2021, for each health post in the study area. Negative binomial regression models were used, with person-time at risk used as an offset and standard errors clustered by health post, to obtain incidence rate ratios (IRRs) and rate differences. Changes in diagnoses were estimated from the administration weeks to each of the three weeks post- administration within the same population. Injury rates were used as a negative control outcome to assess potential unmeasured confounding.

**Results:**

Although SMC was administered during peak malaria transmission weeks within each cycle, both uncomplicated and severe malaria rates remained high through December, following the fourth and final round of SMC. There was a substantial reduction in infection rates in the 3 weeks post SMC, with gradual increases in rates across the three weeks. The rates of uncomplicated and severe malaria per 1000 person-weeks in the administration weeks were 8.5 (95% CI 7.0 to 10.1) and 0.31 (95% CI 0.22 to 0.40), respectively. Uncomplicated malaria rates were lower by 41%, 95%CI (31–50%), 34% (23–43%) and 22% (12–31%) in the first, second and third weeks after administration, respectively. Severe malaria rates declined by 47% (21–64%), 47% (31–59%) and 34% (17–47%) in the three weeks post-administration. Injury rates, the negative control outcome, did not change significantly across the three weeks.

**Conclusion:**

In programme settings, at the population level, SMC administration was associated with a substantial reduction in uncomplicated and severe malaria, though this effect was limited to the immediate weeks following administration. The gradual increase in malaria rates by the third week suggests a shorter duration of protection than previously observed. Extending the areas where 5 rounds of distribution occur may be necessary to effectively prevent malaria infections in regions with a longer transmission season. Regular evaluation of local malaria trends and impact of SMC can help further tailor and optimize SMC programmes for specific regional contexts.

**Supplementary Information:**

The online version contains supplementary material available at 10.1186/s12936-025-05597-y.

## Background

Sub-Saharan Africa accounts for a large proportion of the under 5 mortality world-wide [1, 2]. Malaria is among the leading causes of illness and death in children in low- and middle-income countries [3]. One strategy to prevent malaria infection and reduce childhood mortality is the administration of seasonal malaria chemoprevention (SMC) for children under five years of age. SMC is administration of a full treatment course of antimalarial drug, i.e. therapeutic doses of sulfadoxine-pyrimethamine (SP) and smodiaquine (AQ) during the malaria transmission season in areas with highly seasonal malaria transmission [4, 5].

Following the World Health Organization (WHO) policy recommendation in 2012, 13 countries in Africa had adopted SMC with varying scales of implementation as of 2020 [4, 5], with this number growing to 18 by 2023 [6]. SMC is typically administered in four rounds, spaced four weeks apart, between July and October, with varying levels of coverage and uptake by village. Given recent recommendations that include flexibility in age group, dosage and frequency of treatment based on local conditions [7], some regions administer 3 to 5 rounds, depending on the local malaria transmission dynamics [7].

Since its implementation, SMC has been shown to prevent clinical malaria and all-cause mortality [8]. Although initial evidence from randomized controlled trials (RCTs) and previous evaluations suggests that SMC is effective [9, 10], further research is needed to assess whether it remains effective over time across various ecological and epidemiological settings including different malaria transmission intensity, drug adherence, and local healthcare infrastructure. Studies suggest that targeted interventions for reducing malaria transmission need to consider the local environmental and climatic factors to be effective [11], making context-specific evaluations critical. For instance, Burkina Faso has unique transmission features like incidence rebounds, intra-annual seasonality, and significant heterogeneity in malaria patterns [11]. Malaria cases in Burkina Faso have been increasing since 2016, with over 11 million cases reported in 2020, resulting in nearly 4000 deaths [11]. Evaluating trends in such specific regions can reveal aspects of malaria transmission and the impact of SMC that may not be as evident in other areas.

Short-term assessments of the effects of SMC have typically been limited to RCTs in which administration is highly controlled [12]. Some studies have compared malaria infection in the years before (prior to 2014) and after SMC implementation [10]. Other studies have focused on the safety and effectiveness of SMC during its initial years of implementation and expansion [13]. Evaluating population-level data in real-world program settings is essential for understanding the effectiveness and public health impact of SMC in the context of operational challenges, such as varying treatment coverage, increasing drug resistance, and the presence of other malaria interventions and vector control strategies. A detailed short-term analysis of SMC's effectiveness, including how and when it works and the duration of its protection, is crucial for optimizing its implementation. Ongoing monitoring and evaluation of SMC distribution, along with analyses of surveillance data, can provide valuable insights into changes in its population-level impact, helping to monitor resistance to treatment, optimize the programme, allocate resources effectively, and adjust policies as needed.

Additionally, SMC administration has previously been associated with reductions in hospitalization and deaths [8]. Evaluating the effectiveness of SMC in preventing severe malaria cases helps determine whether SMC not only reduces overall malaria rates but also lowers the incidence of severe disease in the population. This assessment can provide insights into whether SMC continues to effectively address these issues. It will also help determine if additional strategies are needed to prevent severe cases that could lead to hospitalizations and deaths. This is particularly important in resource-constrained regions with limited health infrastructure.

The short-term effects of SMC in the weeks following administration were evaluated using real-world healthcare data for malaria diagnoses among children under five from government-run primary healthcare facilities in Nouna, Burkina Faso, census data from a cluster randomized trial of 278 villages in Burkina Faso [14] and SMC administration dates. The objective of this study was to: (1) describe trends in uncomplicated and severe malaria incidence rates in the presence of SMC, and (2) evaluate the effect of SMC by comparing malaria rates during administration weeks to those in the three weeks following SMC administration in Burkina Faso.

## Methods

### Study setting and population

Health posts in the Community Health with Azithromycin Trial (CHAT) study area (n = 52) were included in the analysis [14]. CHAT was a cluster RCT of 285 villages, designed to evaluate the efficacy of mass and targeted azithromycin strategies for reducing child mortality, and was not related to SMC [14]. It was conducted for 3 years (2019–2023) in the rural northwestern district of Nouna in Burkina Faso and covered both the Nouna Health and Demographic Surveillance Site (HDSS), and the surrounding non-HDSS area [15]. The census performed for the CHAT trial was used to generate the population denominator for this study. CHAT’s methods, including a map of the study region and size of the study, have been described in detail previously [14, 15].

According to a previous study, the median rate of healthcare visits in the CHAT villages in 2020 was 6.7 per 100 child-months. The most common reasons for visits were pneumonia (37.5%), malaria (25.1%) and diarrhoea (9.1%) [16]. This analysis included data on uncomplicated and severe malaria diagnoses from 2021 for children aged 3–59 months, as reported by the health posts in the CHAT study area.

The study was reviewed and approved by the Institutional Review Boards at the University of California, San Francisco and the Comité National d’Ethique pour la Recherche (National Ethics Committee of Burkina Faso) in Ouagadougou, Burkina Faso. This study also adheres to all relevant aspects of the STROBE guidelines for observational studies, including study design, methods, statistical analysis and reporting of results.

### Data sources and measures

Three data sources were used for this analysis: (1) primary healthcare surveillance, (2) CHAT census data, and (3) Burkina Faso’s National Malaria Control Programme (NMCP) data.

#### Primary healthcare surveillance

This data was collected from government-led primary healthcare facilities. In Burkina Faso, children under five years of age receive free care at Centres de Santé et de Promotion Sociale (CSPS), which are government-run, nurse-led primary healthcare facilities offering basic preventive and treatment services, including antenatal care, postnatal care, and vaccinations for children. Passive surveillance was conducted at each CSPS (health post) in the CHAT study area. In all facilities, data were extracted from ledgers issued by the Ministry of Health and entered into an electronic data capture form [16]. Each visit by a child under five was recorded, including the reason for the visit (incl. fever, diarrhoea, malnutrition), the village of residence, the child’s age and sex, the diagnosis (incl. malaria, pneumonia), the treatment provided (incl. antibiotics, antimalarial agents.), and the timing of the visit (e.g., first versus follow-up visit). Clinic visit counts for uncomplicated and severe malaria cases were used to determine diagnosis rates while injury rates were used as negative control. Malaria was diagnosed using a rapid diagnostic test (RDT) when available or based on symptoms during stockouts, while severe malaria diagnoses were made according to WHO criteria [17]. Injuries included various types, such as burns, wounds and bites. The surveillance data was restricted to villages in Nouna where the census was conducted and to the year 2021, for which SMC administration data were obtained.

#### Community Health with Azithromycin Trial (CHAT) data

In this trial, a census was conducted every 6 months documenting births, deaths, pregnancies, and migration. The census data from the RCT was used because it was a door-to-door population census conducted during the same time frame as the clinic visits, making it the most recent available data. The 2021 census data was used as denominator to derive the number of children in each health post, which was then used to calculate malaria rates.

#### National Malaria Control Programme (NMCP) data

This data was used to obtain SMC administration dates and the corresponding epidemiological (epi) weeks for each round of treatment. Epi week or a CDC week is a standardized method of counting weeks to allow comparison of data year after year [18]. Each epi week begins on a Sunday and ends on Saturday [18]. In 2021, SMC was administered on July 6–9 (epi-week 27), August 3–6 (epi-week 31), August 31–September 2 (epi-week 35) and September 28–October 1 (epi-week 39). During each round, SMC was administered to the entire study area during the same epi week over a 3–4 day period. Typically, in Burkina Faso, SMC is administered through door-to-door campaigns by community health workers [19]. Coverage for each health post was calculated as the number of children aged under 5 that were treated divided by the number of total children aged under 5 in the CSPS catchment area.

### Study design and statistical analysis methods

For the first objective of this study, malaria trends were described by estimating malaria diagnosis rates for each of the 52 epi weeks in 2021. For the second objective, a quasi-experimental approach was used, employing a one-group pretest–posttest design with multiple follow-up measurements [20]. Malaria rates during the administration weeks were compared with rates in the first, second-, and third weeks following administration. These three weeks after administration as the post-intervention period were selected because SMC is administered every four weeks, with the fourth week marking the administration of a new cycle [4, 5]. Additionally, the effectiveness of SMC has previously been shown to last about 28 days [13, 21, 22], Therefore, this setup of administration and post-administration weeks effectively enables us to evaluate its short-term impact on infections in the population.

Malaria diagnoses over the year were aggregated to counts by epi week for each health post (CSPS) in the study area. Census data were to calculate the total number of children in each CSPS for the year, which was used to calculate person-week. The census and surveillance data at the health post level were joined by CSPS. For each CSPS, weekly diagnoses count of malaria were included in a longitudinal format. Malaria incidence rates and corresponding 95% confidence intervals were estimated for each of the 52 epi weeks, including the periods during and after SMC distribution. Uncomplicated and severe malaria incidence rates for 2021 were plotted, highlighting the malaria season (June-December) and the SMC administration weeks, to visualize diagnosis patterns and any changes following SMC administration.

For the pre-post comparison, a variable was created to indicate the epi weeks of the four rounds of SMC administration in 2021, with indicators for administration weeks and the first, second-, and third-weeks post administration. The administration week served as reference against which the rates for each week post administration were compared. Approximately 10% of the epi weeks in 2021 across all health posts did not have recorded counts for uncomplicated and severe malaria diagnoses. Given the limited amount of missing data and set of covariates, a complete-case analysis was conducted.

A negative binomial regression model was used with person-weeks as an offset and standard errors clustered by health post to account for clustering by health post. Marginal effects, expressed as incidence rate ratios (IRR) and incidence rate differences (IRD), were calculated across all health posts by comparing malaria rates during the four rounds of SMC administration weeks with those in the first, second-, and third-weeks post administration. In these models, the month of SMC administration was included as a covariate to account for seasonal changes, as the month is a predictor of outcomes that could potentially influence these rates. Since this assessment compares rates in the same population during and after administration, time-invariant factors and confounders are accounted for by design [23].

Additionally, since all communities were treated and there was no exposure control group, a negative control outcome was used to assess potential unmeasured confounding from other factors that may change over time [24, 25]. Injury rate was used as a negative control outcome in a sensitivity analysis, as it would share the same potential sources of bias (given that these were reported by the same facilities using the same process) and is an outcome that cannot be related to treatment (SMC) [24, 25].

SAS 9.4 (SAS Institute, Cary, NC) was used for data cleaning and for generating the dataset for analysis. Stata version 14.2 (StataCorp, College Station, TX) was used for all other analyses.

## Results

Across the 52 health post catchment areas, the average number of children was 2447, with an average age of 40.9 months (3.4 years). Over the year, the average rates of malaria, severe malaria, and injury were 5.3 (95% CI 4.4 to 6.1) cases per 1000 person-weeks, 0.15 (95% CI 0.12–0.18) cases per 1000 person-weeks, and 0.15 (95% CI 0.12–0.18) cases per 1,000 person-weeks, respectively. Both uncomplicated and severe malaria rates were highest in the last quarter of the year going up to 8.9 (95% CI 7.3–10.5) cases per 1,000 person-weeks, and 0.26 (95% CI 0.20–0.33) cases per 1,000 person-weeks, respectively. The average SMC coverage was approximately 80%, with coverage estimates for the 4 rounds being 78.6%, 78.3%, 79.6% and 81.2%, respectively Table 1.

**Table 1:** Diagnosis rates per 1,000 person-weeks (95% CI) among children under 5 at CSPS/health posts in Nouna, BurkinaFaso, by calendar quarter

Rates per 1000 person-week (95%CI)	Uncomplicated malaria	Severe malaria	Injury (negative control)
Overall	5.3 (4.4 to 6.1)	0.15 (0.12 to 0.18)	0.15 (0.12 to 0.18)
Quarter 1 (Jan–Mar)	5.8 (4.9 to 6.8)	0.13 (0.09 to 0.16)	0.15 (0.12 to 0.19)
Quarter 2 (Apr–Jun)	2.4 (1.9 to 2.9)	0.04 (0.02 to 0.06)	0.16 (0.12 to 0.19)
Quarter 3 (July–Sept)	4.3 (3.5 to 5.1)	0.17 (0.11 to 0.23)	0.14 (0.1 to 0.18)
Quarter 4 (Oct–Dec)	8.9 (7.3 to 10.5)	0.26 (0.2 to 0.33)	0.14 (0.1 to 0.17)

CSPS: Centres de Santé et de Promotion Sociale

**Fig. 1:**
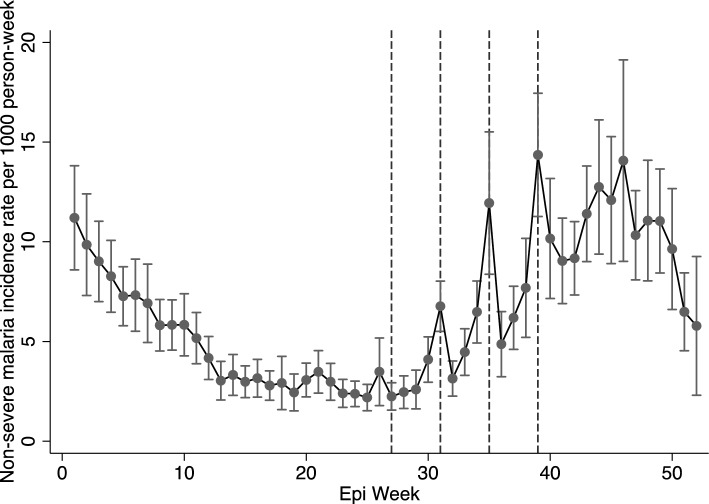
Uncomplicated malaria incidence rates in 2021 in the presence of SMC. Broken lines represent SMC administration. In 2021, SMC was administered in four rounds: July 6−9 (epi-week 27), August 3–6 (epi-week 31), August 31–September 2 (epi-week 35) and September 28–October 1 (epi-week 39). These rates are for children under 5

**Fig. 2:**
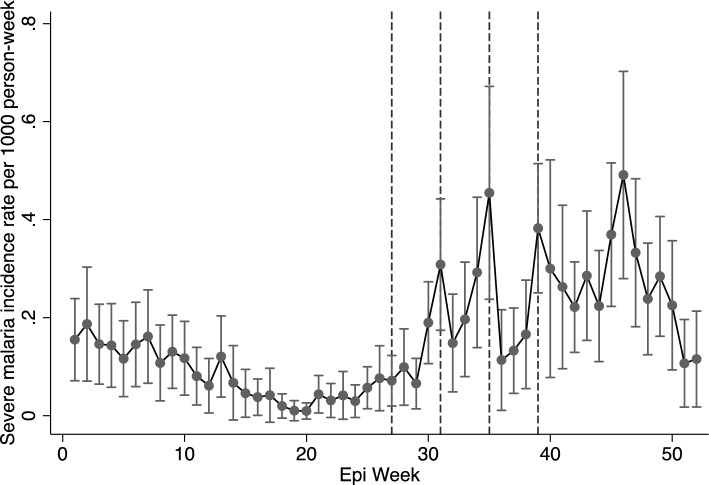
Severe malaria incidence rates in 2021 in the presence of SMC. Broken lines represent SMC administration. In 2021, SMC was administered in four rounds: July 6–9 (epi-week 27), August 3–6 (epi-week 31), August 31–September 2 (epi-week 35) and September 28–October 1 (epi-week 39). These rates are for children under 5

The uncomplicated malaria rates for the entire year, including the malaria transmission season in 2021, are shown in Fig. 1. Malaria rates declined from January to March (epi weeks 1–10), began increasing towards the end of July (epi week 30), and remained high through the end of December (epi week 50). Malaria rates remained high after the fourth and final round of SMC, peaking between late September and early December (Epi weeks 39–49), with incidence rates ranging from 10 to 15 cases per 1000 person-weeks. Rates of severe malaria diagnosis showed similar trends, increasing from the end of July through the end of the year (Epi weeks 30–52) (Fig. 2).

Injury diagnosis rates remained consistent throughout the year, as expected (Supplementary Fig. 1).

**Fig. 3:**
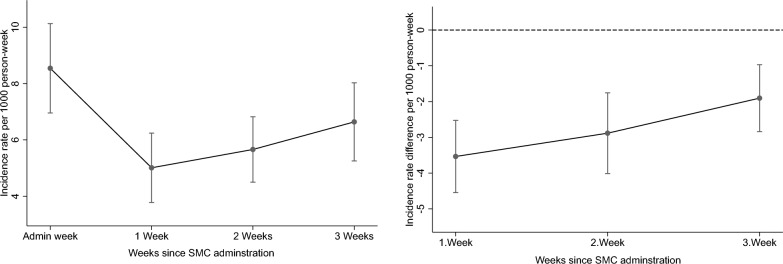
Incidence rate and rate difference of uncomplicated malaria in the weeks following SMC compared toadministration weeks among children under 5 years. (**a**) Incidence rate per 1,000 person-weeks with 95% CI by week since SMC administration (administrationweek, 1 week, 2 weeks, 3 weeks). (**b**) Incidence rate difference per 1,000 person-weeks with 95% CI by week since SMC administration

Table 2 shows the IRR and IRD for the change in rates during the 3 weeks post administration. There was a substantial reduction in malaria rates during the 3 weeks post-SMC, but this reduction was not linear (Table 2; Figs. 3a and b). Compared to rates during the administration weeks, uncomplicated malaria rates were lower by 36% (95% CI 24–45%), 37% (95% CI 27–45%), and 23% (95% CI 12–33%) in the first, second, and third weeks after administration, respectively (Table 2; Figs. 3a and b). After each round of administration and across all rounds, malaria rates declined significantly in the first week and increased again over the three weeks post- administration (Figs. 1; 3a and b). For the control outcome, injury, diagnosis rates remained unaffected during the post-SMC administration period, as expected (Supplementary Figs. 2a and b).

**Table 2:** Incidence rate, rate ratio and rate difference of uncomplicated and severe malaria during SMC administration weeks and the first–second- and third weeks post administration

Weeks post SMC administration	Incidence rate per 1000 person-week (95%CI)	Incidence rate ratio (95%CI)	Incidence rate difference per 1000 person-week (95%CI)
Uncomplicated malaria			
0 (Admin week)	8.5 (7.0 to 10.1)	Ref.	Ref.
1	5.0 (3.8 to 6.2)	0.59 (0.50 to 0.69)	− 3.5 (− 4.5 to − 2.5)
2	5.7 (4.5 to 6.8)	0.66 (0.57 to 0.77)	− 2.9 (− 4.0 to − 1.8)
3	6.6 (5.3 to 8.0)	0.78 (0.69 to 0.88)	− 1.9 (− 2.8 to − 1.0)
Severe malaria			
0 (Admin week)	0.31 (0.22 to 0.40)	Ref.	Ref.
1	0.17 (0.08 to 0.26)	0.53 (0.36 to 0.79)	− 0.14 (− 0.21 to − 0.08)
2	0.17 (0.1 to 0.23)	0.53 (0.41 to 0.69)	− 0.14 (− 0.2 to − 0.09)
3	0.21 (0.14 to 0.27)	0.66 (0.53 to 0.83)	− 0.10 (− 0.16 to − 0.05)
Injury (negative control)			
0 (Admin week)	0.16 (0.08 to 0.23)	Ref.	Ref.
1	0.11 (0.07 to 0.15)	0.68 (0.40 to 1.15)	− 0.05 (− 0.13 to 0.03)
2	0.17 (0.11 to 0.23)	1.06 (0.66 to 1.72)	0.01 (− 0.07 to 0.09)
3	0.14 (0.09 to 0.20)	0.89 (0.51 to 1.55)	− 0.02 (− 0.1 to 0.07)

Estimates were obtained from models that adjusted for the month of administration. Analyses include children under 5

**Fig. 4:**
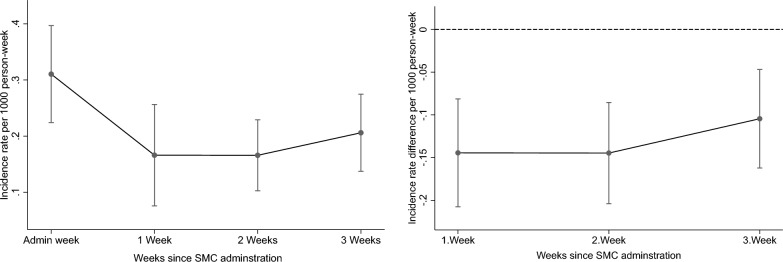
Incidence rate and rate difference of severe malaria in the weeks following SMC compared to administrationweeks among children under 5 years. (**a**) Incidence rate per 1,000 person-weeks with 95% CI by week since SMC administration (administrationweek, 1 week, 2 weeks, 3 weeks). (**b**) Incidence rate difference per 1,000 person-weeks with 95% CI by week since SMC administration

This trend was similar for severe malaria diagnoses, with a modest decline in rates during the first week (IRR 0.59, 95% CI 0.41 to 0.86), an even greater decline in the second week (IRR 0.49, 95% CI 0.35 to 0.68), and a slight increase in rates during the third week after administration (IRR 0.75, 95% CI 0.60 to 0.95) (Table 2; Figs. 4a and b).

## Discussion

Healthcare surveillance data on malaria diagnoses were used to evaluate the short-term effectiveness of SMC under routine programme conditions in Burkina Faso. Findings indicate that although SMC was administered during the 4 months of the high malaria transmission season, malaria rates remained elevated through December. This suggests that extending or shifting the SMC schedule could be beneficial in regions with similarly prolonged transmission seasons as it appears that transmission starts earlier in June, when rates are still relatively low, and stops in October, when rates are high. While both uncomplicated and severe malaria rates declined following SMC administration, they gradually increased by the third week began to rise by the third week. This could indicate that the protective effect of SMC under routine programme conditions may have become shorter at the population level than previously observed.

SMC administration dates were aligned with malaria peak weeks within each round and covered the majority of the malaria transmission season. Since 2014, SMC has been implemented in Nouna, Burkina Faso in four monthly rounds during the rainy season, from July to October [26], while the dry season spans from October to May [27]. Recently, some regions, particularly those with known high-transmission seasons, are being provided with a fifth round of SMC [28]. Findings from this analysis indicate that malaria rates remained high in November and December in this region, which was originally considered to require only four rounds. This suggests that extending the areas where 5 rounds are distributed may be necessary to adequately prevent malaria infections through the high-risk period. Alternatively, regions facing logistical constraints in extending cycles or higher risks of resistance, but experiencing similar transmission patterns, could consider adjusting the SMC schedule to target the weeks with the highest malaria transmission, particularly those later in the year.

Instances where the malaria season extends into the dry season towards the end of the year have been observed in other settings. For example, a study in Mali found that malaria incidence was high past the end of the rainy season in October and remained high until December [29]. Similarly, another time-series analysis found that malaria infections peaked in December [30]. Ongoing malaria transmission can persist into the dry season, with the strongest risk factors in this period being the interrupted use of insecticide treated nets, housing structure and travel history [31]. Factors such as ability of the *Plasmodium falciparum* parasite to adapt and survive longer in host [32], persistence of vector breeding sites, and decline in the use of malaria prevention tools towards the end of the rainy season have previously been suggested as reasons for this extension of transmission into the dry season [29–31].

Given the variations in the length of the malaria transmission season [33], areas with extended transmission may require additional cycles and further optimization of SMC programmes. Careful consideration and assessment of the local malaria epidemiology is crucial for determining the appropriate number of doses and timing of treatment in specific regions. Previous RCTs have shown that a fifth round of SMC is both useful [12] and effective [34]. While some evidence suggests that a fifth round would be beneficial and acceptable to caregivers and stakeholders at national and regional levels [26, 35], more context-specific assessments are needed to evaluate its impact on malaria infection, mortality, and the development of antimalarial resistance [26] in various settings. Additionally, the logistical and financial implications of extending SMC administration, including increased costs, workload and the need for further enhanced monitoring for drug resistance, must be considered when scaling up in regions that previously received fewer doses.

Compared to administration weeks, rates of uncomplicated and severe malaria declined significantly in the first, second, and third weeks post-administration. This sharp decline following administration is consistent with findings from an RCT, which showed markedly lower malaria incidence immediately after each SMC course [12]. This indicates that the high drug efficacy of the SMC treatment combination [12, 22] continues to be apparent under routine operational settings in which there are less optimal conditions such as gaps in coverage. However, the consistent rise in both uncomplicated and severe malaria rates from the third to the fourth week post-administration of each round suggests that the effect of SMC may be becoming shorter than previously noted. Previous studies have shown that the effectiveness of SMC was highest within the first 28 days after administration [13, 21, 22], which was a short duration but slightly longer than that observed here. The shorter duration observed here, compared to that documented in RCT [36], may indicate that the period of protection could be even shorter at the population level in areas with gaps in coverage. Furthermore, this may signal a growing resistance to SP, as shorter duration of protection of chemoprophylaxis has been previously associated with increasing resistance [37, 38]. Given concerns that the benefits of SP may be undermined by drug resistance [10, 39–41], further investigation into the protection period at the population level across different settings is warranted. The risk of anti-malaria drug resistance (for both sulfadoxine-pyrimethamine [SP] and amodiaquine [AQ]) also necessitates a robust monitoring strategy that tracks the prevalence of resistance markers [42].

The short duration of protection also underscores the importance of timing each round of administration to align with peak malaria transmission periods. The timing of each round of SMC should continue to be aligned with the duration of protection [36]. The effectiveness of SMC in malaria prevention and control is influenced by its efficacy, coverage level, and the duration of protection [40]. A modelling study has shown that the duration of protection of SMC is more critical for its public health impact in preventing disease than the drug’s efficacy (parasite killing effect) [43]. Therefore, for chemoprevention, as opposed to treatment, preventing infection throughout the high-risk period and between cycles is more important than high curative efficacy [43]. However, SMC currently appears to be highly effective for only few weeks post-administration. Based on previous studies, maintaining high coverage level remains crucial for adequate population-level protection [10, 13]. These trade-offs should be explored and considered in future selections or developments of SMC drug profiles or alternative formulations to optimize drug properties, maximize public health impact, and potentially minimize the need for multiple doses, given the operational challenges in maintaining high coverage and concerns about resistance to SP.

Previous studies have found that SMC reduces severe outcomes, including hospitalization and death from all-causes [8]. In this analysis, both uncomplicated and severe malaria rates decreased in the weeks following SMC administration, suggesting that SMC remains an effective strategy for reducing the incidence of severe malaria in populations. Additionally, other research has demonstrated that SMC significantly reduced malaria-specific mortality [13], which is among the leading causes of death in children in low- and middle-income countries [3]. While SMC primarily reduces malaria infections, SP also has weak antimicrobial/antibiotic properties that may help prevent other infections [44, 45]. Previous research indicates that SP’s effectiveness in other chemoprevention programmes, such as intermittent preventive therapy for malaria during pregnancy, may involve malaria-independent pathways [46]. Future studies could investigate whether SMC helps reduce illnesses and death from other infections as part of its effect on all-cause mortality or whether its effect is predominantly through prevention of severe malaria outcomes.

This study has some limitations. First, in the absence of an unexposed control group and limited available covariates, the possibility of confounding cannot be fully excluded. However, the use of a negative control outcome, along with comparison of weekly malaria rates within short intervals before and after each SMC cycle, helps minimize the likelihood that other factors could simultaneously change after each round of administration and have a large influence on the outcomes, given that population-level factors tend to change gradually over time. The inclusion of multiple time points/observations in the pre- and post-treatment periods between and across cycles allows the underlying trend of infections to be partially accounted [23]. Additionally, the longitudinal assessment of rates in the same population over time prevents bias from time-invariant factors at the population level, which is a key strength of the pre-post comparisons [23].

Second, there is a potential for misclassification in diagnosing malaria cases, which could affect the weekly malaria count estimates. Due to the absence of data on RDT stockouts or which diagnoses were made based on symptoms versus RDT, a separate analysis of these diagnoses was not possible. However, the standard practice is to use RDTs, and any potential misdiagnoses are expected to be randomly distributed across the weeks, given the consistency in case definitions over these short time periods. Similarly, although diagnoses of severe malaria may vary by facility resources and training, there are unlikely to be related to SMC administration weeks as distribution was uniform, and all facilities were combined for analyses. Additionally, any errors in data entry can also lead to misclassification.

Therefore, any misclassification is likely non-differential between the comparison weeks and would likely attenuate estimates due to random mixing. Another potential limitation is that SMC administration could lead to an artificial increase in reported cases during the SMC week, particularly if fever cases are referred to health posts during household visits. However, with four administrative weeks across 51 facilities, the population-level analysis is expected to help average out short-term fluctuations, while the overall trend observed across multiple weeks and facilities helps mitigate any localized bias. Additionally, surveillance data may be affected by underreporting. However, it provides large-scale, real-time data that allows for effective monitoring of disease trends in populations and is, therefore, valuable for assessing the need for and effect of interventions [47].

Lastly, these are population-level analyses. It is not known whether individual children who had malaria infection received SMC treatment. However, population-level analyses help evaluate the overall public health benefits of treatment, including potential indirect benefits to untreated individuals resulting from herd effects [48]. Given that this study analysed malaria diagnoses reported by health posts in Nouna, Burkina Faso, the findings can be generalized to this region and other settings that share similar malaria profiles and transmission patterns.

## Conclusion

The findings indicate that SMC administration was associated with a substantial reduction in both uncomplicated and severe malaria, but this effect was evident only in the immediate weeks following administration. This suggests that while SMC remains highly effective shortly after administration, its duration of protection at the population level may have become shorter than previously observed. Although SMC administration was aligned with peaks in infection and the protection period within each round, transmission patterns suggest that expanding the geographic areas covered by the 5 rounds of SMC may be necessary to effectively prevent malaria in regions with extended transmission seasons. Additionally, findings highlight the need for continuous, localized monitoring of malaria rates to further tailor and optimize SMC programmes to specific regional contexts.

## Supplementary Information

Supplementary Material 1: Figure 1- Injury incidence rates in 2021 in the presence of SMC. Broken lines represent SMC administration. In 2021, SMC was administered in four rounds: July 6- 9, August 3 - 6, August 31- September 2and September 28- October 1. These rates are for children under 5. Figure 2a and 2b- Incidence rate and rate difference of injuryin the weeks following SMC compared to administration weeks. Analyses include only children under 5

## Data Availability

The data for this study is available upon request.
