# Supplementary material for: Trends in uncomplicated and severe malaria following seasonal malaria chemoprevention administration in Nouna, Burkina Faso: a quasi-experimental pre-post study
Source: Malar J. 2025 Nov 6;24:376. doi: 10.1186/s12936-025-05597-y (PMC12590836; doi:10.1186/s12936-025-05597-y)
Supplement: Supplementary file 1 — Supplementary Material 1: Figure 1- Injury incidence rates in 2021 in the presence of SMC. Broken lines represent SMC administration. In 2021, SMC was administered in four rounds: July 6- 9, August 3 - 6, August 31- September 2and September 28- October 1. These rates are for children under 5. Figure 2a and 2b- Incidence rate and rate difference of injuryin the weeks following SMC compared to administration weeks. Analyses include only children under 5 [file 12936_2025_5597_MOESM1_ESM.docx]

**Supplementary Material**

**Supplementary Figure 1**- Injury incidence rates in 2021 in the presence of SMC

**Supplementary Figure 2a and 2b-** Incidence rate and rate difference of injury (negative control) in the weeks post SMC compared to administration weeks

Note: Analyses include children under 5
